# Brigatinib versus other second-generation ALK inhibitors as initial treatment of anaplastic lymphoma kinase positive non-small cell lung cancer with deep phenotyping: study protocol of the ABP trial

**DOI:** 10.1186/s12885-021-08460-w

**Published:** 2021-06-28

**Authors:** Petros Christopoulos, Farastuk Bozorgmehr, Lena Brückner, Inn Chung, Johannes Krisam, Marc A. Schneider, Albrecht Stenzinger, Regina Eickhoff, Daniel W. Mueller, Michael Thomas

**Affiliations:** 1 Department of Thoracic Oncology, Thoraxklinik at University Hospital of Heidelberg, Röntgenstraße 1, 69126 Heidelberg, Germany; 2 Translational Lung Research Center Heidelberg TLRCH, Member of the German Center for Lung Research DZL, Im Neuenheimer Feld 156, 69120 Heidelberg, Germany; 3 University Hospital of Heidelberg, Institute of Medical Biometry and Informatics, Im Neuenheimer Feld 130.3, 69120 Heidelberg, Germany; 4 Translational Research Unit (STF), Thoraxklinik at University Hospital of Heidelberg, Röntgenstraße 1, 69126 Heidelberg, Germany; 5 University of Heidelberg, Institute of Pathology, Im Neuenheimer Feld 224, 69120 Heidelberg, Germany; 6 Institut für Klinische Krebsforschung IKF GmbH am Krankenhaus Nordwest, Steinbacher Hohl 2-26, 60488 Frankfurt am Main, Germany

**Keywords:** Non-small cell lung cancer, Anaplastic lymphoma kinase, ALK^+^ NSCLC, Tyrosine kinase inhibitors (TKI), Brigatinib, Resistance mutations, Molecular risk

## Abstract

**Background:**

Availability of potent anaplastic lymphoma kinase (ALK) tyrosine kinase inhibitors (TKI) has pushed the median survival of ALK^+^ non-smallcell lung cancer (NSCLC) patients to over five years. In particular, second-generation ALK TKI have demonstrated superiority compared to the first-generation compound crizotinib and are meanwhile standard first-line treatment. However, clinical courses of individual patients vary widely, with secondary development of drug resistance and intracranial progression remaining important problems. While these limitations highlight the need for better disease monitoring and additional therapeutic tools, molecular tumor features are increasingly recognized as crucial determinants of clinical outcome. This trial aims to optimize management of ALK^+^ NSCLC by analyzing the efficacy of second-generation ALK inhibitors in conjunction with deep longitudinal phenotyping across two treatment lines.

**Methods/design:**

In this exploratory prospective phase II clinical trial, newly diagnosed ALK^+^ NSCLC patients will be randomized into two treatment arms, stratified by presence of brain metastases and ECOG performance status: brigatinib (experimental arm) vs. any other approved second-generation ALK TKI. Tumor tissue and blood samples will be collected for biomarker analysis at the beginning and throughout the study period to investigate baseline molecular tumor properties and analyze the development of acquired drug resistance. In addition, participating investigators and patients will have the possibility of fast-track molecular tumor and ctDNA profiling at the time of disease progression using state-of-the-art next-generation sequencing (NGS), in order to support decisions regarding next-line therapy.

**Discussion:**

Besides supporting therapeutic decisions for enrolled patients, the ABP trial primarily aims to deepen the understanding of the underlying biology and facilitate development of a framework for individualized management of ALK^+^ NSCLC according to molecular features. Patients with low molecular risk and the perspective of a “chronic disease” will be distinguished from “high-risk” cases, molecular properties of which will be utilized to elaborate improved methods of non-invasive monitoring and novel preclinical models in order to advance therapeutic strategies.

**Trial registration:**

Clinicaltrials.gov, NCT04318938. Registered March 182,020, *https://www.clinicaltrials.gov/ct2/show/NCT04318938*

Eudra-CT, 2019–001828-36. Registered September 302,019, *https://www.clinicaltrialsregister.eu/ctr-search/search?query=2019-001828-36*

## Background

Non-small cell lung cancer (NSCLC) is the leading cause of cancer-related mortality worldwide with most patients diagnosed at an advanced, incurable stage [1–3]. Over the past years, advanced molecular diagnostics have revealed distinct driver molecular alterations in about 15–20% of these tumors, which also constitute particular therapeutic susceptibilities. Targeted treatment with tyrosine kinase inhibitors (TKI) has consistently demonstrated superior efficacy and tolerability compared to conventional chemotherapy in eligible patients and is meanwhile the standard first-line option.

*ALK* (anaplastic lymphoma kinase)-positive tumors comprise approximately 5% of metastatic NSCLC, mainly adenocarcinomas of younger, non-smoker patients [4, 5], and are unique in many aspects: They typically have a lower number of genetic alterations compared to other NSCLC subtypes, as partly reflected by a mean tumor mutational burden (TMB) uniquely below 3 mut/Mb [6, 7]. ALK^+^ NSCLC patients require the most sophisticated management at present, i.e., high-level expertise and close cooperation between medical oncology, interventional pneumology, radiology, thoracic surgery, radiation oncology, as well as molecular pathology over several years, and enjoy the best outcome. Even the first-generation compound crizotinib results in longer overall survival (OS) for ALK^+^ NSCLC patients compared to their TKI-treated EGFR^+^ counterparts (for example > 46 months in the PROFILE 1014 study vs. 39 and 32 months in the two arms of the FLAURA trial), while sequential ALK TKI, particularly with upfront administration of second-generation inhibitors such as alectinib, confer a median OS over five years, which is certainly one of the greatest successes in modern thoracic oncology [6, 8–10]. First-line administration of the newer second-generation inhibitor brigatinib has produced comparable results to alectinib in the randomized phase III ALTA-1 L trial [11, 12], and both substances are currently initial treatments of choice [13, 14]. An important advantage of both compounds is enhanced intracranial efficacy, with brain overall response rates (iORR) of approximately 80% and rates of intracranial progression < 10% per year, compared to 30–50% and approximately 20% with the first-generation inhibitor crizotinib, respectively. Very recently, interim results from the CROWN trial, comparing lorlatinib against crizotinib in the first line, have been reported [15]. With a median follow-up of 18 months, data are still immature, but the hazard ratio (HR) of 0.28 for progression-free survival (PFS) in favor of the third-generation TKI lorlatinib indicates that this compound will probably also play a role as upfront therapy in the future. At the same time, the generally favorable outcome of ALK^+^ NSCLC facilitates identification of special patient subsets with earlier treatment failure and higher risk of death. Currently, high-risk ALK^+^ disease comprises three main groups: Tumors with the *EML4-ALK* fusion variant 3 (V3), whose shorter structure lowers TKI sensitivity and increases metastatic potential [16–20], tumors with *TP53* mutations, either at initial diagnosis [21, 22] or at the time of TKI failure [23], and “double-positive patients”, who have the worst outcome [22], as also recently demonstrated in the context of the randomized phase 3 ALTA-1 L trial [24]. Importantly, repeat molecular profiling based on tissue or liquid rebiopsies at the time of disease progression can identify actionable resistance mutations and guide individualized selection of next-line targeted therapies with higher clinical benefit for the patient [25, 26, 27].

The aim of this trial is to optimize management of ALK^+^ NSCLC by analyzing the efficacy of brigatinib and other ALK TKI in conjunction with deep clinical and molecular patient phenotyping across two treatment lines.

## Methods/design

### Study design

This is a prospective, randomized, open-label, multicenter phase II trial comparing brigatinib vs. other second-generation ALK inhibitors as first-line therapy for ALK^+^ NSCLC in conjunction with deep patient profiling across two treatment lines.

### Study setting

The ABP trial will recruit patients from currently 24 participating centers across Germany over a period of 36 months. Recruitment started in June 2020. A full list of sites is available at clinicaltrials.gov (NCT04318938).

### Study objectives and endpoints

The primary objective of this trial is a descriptive comparison of the efficacy of brigatinib vs. other approved second-generation ALK TKI in first-line treatment of locally advanced or metastatic ALK^+^ NSCLC, as assessed by progression-free survival (PFS1) according to RECIST 1.1 [28]. Secondary objectives are treatment efficacy in the first and second line, assessed by the PFS of second-line therapy (PFS2), time-to-next treatment in the first line (TNT1), second line (TNT2) and until start of the third line (TNT1/2), as well as by the overall survival (OS). Moreover, CNS efficacy of first- and second-line treatment will be evaluated according to RECIST 1.1 regarding iORR, intracranial duration of response (iDOR) and time-to-intracranial progression (iTTP).

Safety in both treatment arms will be evaluated with regard to incidence and severity of adverse events (AE), while quality of life (QoL) will be assessed using the validated questionnaires SF-12 and EORTC-QLQ-BN20 (the latter in case of brain metastases, only).

Exploratory objectives are typing of *ALK* fusion variants, assessment of *TP53* status, profiling of resistance mechanisms at the failure of first-line treatment, and their relationship with efficacy endpoints. Furthermore, the potential clinical utility of cerebrospinal fluid ctDNA analysis will be assessed for cases with isolated intracranial progression.

### Characteristics of participants

A total of 116 adult patients with newly diagnosed ALK^+^ NSCLC, either locally advanced (stage III) and not suitable for curative treatment, or metastatic (stage IV), will be enrolled into this study after written informed consent. The full list of inclusion and exclusion criteria is given in Table 1. *ALK* rearrangement will be determined locally by one or more ALK assays that are approved in Germany and/or have been validated within the German Network for Genomic Medicine, e.g. RNA-based NGS, fluorescence in situ hybridization using the ZytoLight SPEC ALK probe (ZytoVision GmbH, Bremerhaven, Germany) and immunohistochemistry using the D5F3 clone (Roche, Mannheim, Germany) [18, 29–31].

**Table 1:** Inclusion and exclusion criteria

**INCLUSION CRITERIA**	• Fully informed written consent and any locally-required authorization (EU Data Privacy Directive) given by the patient • Male or female ≥18 years of age • Histologically confirmed locally advanced (stage III) and not suitable for curative treatment, i.e. R0 operation or definitive chemo−/radiation, or metastatic (stage IV) ALK^+^ NSCLC NOTE: Documentation of ALK rearrangement confirmed at baseline by a positive result of any ALK assay approved in Germany [i.e. positivity for at least one of the three: immunohistochemistry (IHC), NGS, fluorescence in situ hybridization (FISH)]. Treatment may be started based on a local ALK^*+*^ test result, but subsequent central molecular profiling of the baseline biopsy, incl. determination of *ALK* variant and *TP53* status, should be made possible for all patients. • No prior therapy for metastatic ALK^+^ NSCLC including ALK inhibitors (up to two cycles of chemotherapy as well as cerebral irradiation before inclusion in the study are acceptable) • At least 1 measurable (i.e., target) lesion according to RECIST 1.1 • ECOG performance status ≤2 • Adequate organ function, as determined by: - Total bilirubin ≤1.5x the upper limit of the normal range (ULN) (< 3x the ULN in case of Gilbert’s disease) - Estimated glomerular filtration rate ≥ 30 mL/minute/1.73 m^2^ (calculated by MDRD or any other validated formula) - Alanine aminotransferase/aspartate aminotransferase ≤2.5x ULN NOTE: ≤5x ULN is acceptable in case of liver metastases - Serum lipase ≤1.5x ULN - Platelet count ≥75 × 10^9^/L - Hemoglobin ≥9 g/dL - Absolute neutrophil count ≥1.5 × 10^9^/L • Willingness and ability to comply with scheduled visits and study procedures • Willingness to participate in accompanying research program • Collection of current biopsies during screening must be feasible NOTE: For each patient a FFPE-tumor tissue block must be available for biomarker evaluation. Excisional, incisional or core needle biopsies are appropriate, while fine needle aspirations are insufficient. • Women of childbearing potential must have a negative pregnancy test result within 7 days before randomization. Women must not be breastfeeding. • Females who are postmenopausal for at least 1 year before the screening visit OR are surgically sterile OR, if they are of childbearing potential, agree to practice two effective methods of contraception simultaneously, from the time of signing the informed consent until 4 months after the last dose of study drug, or agree to completely abstain from heterosexual intercourse. Males, even if surgically sterilized (i.e., status post vasectomy), who agree to practice effective barrier contraception during the entire study treatment period and until 4 months after the last dose of study drug, OR agree to completely abstain from heterosexual intercourse.
**EXCLUSION CRITERIA**	• History or presence of pulmonary interstitial disease, drug-related pneumonitis, or radiation pneumonitis at baseline • Uncontrolled hypertension (hypertension must be adequately treated for control of blood pressure upon study entry) • Systemic treatment with strong cytochrome P-450 (CYP) 3A inhibitors, strong CYP3A inducers, or moderate CYP3A inducers or treatment with any investigational systemic anticancer agents, chemotherapy or radiation therapy (except stereotactic radiosurgery or stereotactic body radiation therapy) within 14 days of randomization • Treatment with antineoplastic monoclonal antibodies within 30 days of randomization • Major surgery within 30 days of randomization. Minor surgical procedures are allowed, such as catheter placement or minimally invasive biopsies. • Current spinal cord compression (symptomatic or asymptomatic). Patients with leptomeningeal disease without spinal cord compression may participate. • Significant or uncontrolled cardiovascular disease, specifically including, but not restricted to the following: - If an acute coronary syndrome has ensued in the past six months, successful reperfusion has to be documented and the patient must be asymptomatic - New York Heart Association Class III or IV heart failure within six months prior to randomization - Any history of clinically significant ventricular arrhythmia • Cerebrovascular accident or transient ischemic attack within six months prior to first dose of study drug • Malabsorption syndrome or other gastrointestinal illness or condition that could affect oral absorption of the study drug • Active severe or uncontrolled chronic infection, including but not limited to, the requirement for intravenous antibiotics for longer than two weeks • History of HIV infection. Testing is not required in the absence of history. • Chronic hepatitis B (surface antigen-positive) or chronic active hepatitis C infection. Testing is not required in the absence of history. • Any serious medical condition or psychiatric illness that could, in the investigator’s opinion, potentially compromise patient safety or interfere with the completion of treatment according to this protocol • Known or suspected hypersensitivity to brigatinib or other TKI or their excipients • Life-threatening illness unrelated to cancer • Involvement in the planning and/or conduct of the study (applies to both Takeda staff and/or staff of sponsor and study site) • Patient who might be dependent on the sponsor, site or investigator • Patient who has been incarcerated or involuntarily institutionalized by court order or by the authorities [§ 40 Abs. 1 S. 3 Nr. 4 AMG] • Patients who are unable to consent because they do not understand the nature, significance and implications of the clinical trial and therefore cannot form a rational intention in the light of the facts [§ 40 Abs. 1 S. 3 Nr. 3a AMG] • Legal incapacity or limited legal capacity • Females who are pregnant or breastfeeding • Patients who have symptomatic CNS metastases (parenchymal or leptomeningeal) at screening or asymptomatic disease requiring an increasing dose of corticosteroids to control symptoms within 7 days before randomization. NOTE: If a patient has worsening neurological symptoms due to CNS metastasis, they must have completed local therapy and be neurologically stable (without requiring an increasing dose of corticosteroids or anticonvulsants) seven days before randomization. • Rare hereditary galactose intolerance, total lactase deficiency or glucose-galactose malabsorption

In addition, participants must have at least one measurable site of disease as defined by RECIST 1.1, sufficient ECOG performance status (≤ 2), as well as adequate bone marrow, hepatic and renal function. Participants may not have received prior systemic therapy for ALK^+^ NSCLC, however up to two cycles of chemotherapy as well as stereotactic radiotherapy are allowed to accommodate for the fact that in some cases treatment might be initiated in an urgent manner before availability of molecular results. Further exclusion criteria include pulmonary interstitial disease, pneumonitis, uncontrolled hypertension and cardiovascular disease, significant infections or any other medical condition that may potentially compromise patient safety or hamper study completion.

### Study procedures

Patients will be randomized into the experimental (brigatinib) or standard arm in a 1:1 ratio. The variance minimization method will be used for patient allocation, adjusting for the confounders brain metastases (presence vs. absence of brain metastases at baseline) and ECOG performance status (0–1 vs. 2) in order to ensure better comparability between intervention groups. Overall, 58 patients will be enrolled per treatment arm.

#### Treatment

**Fig. 1:**
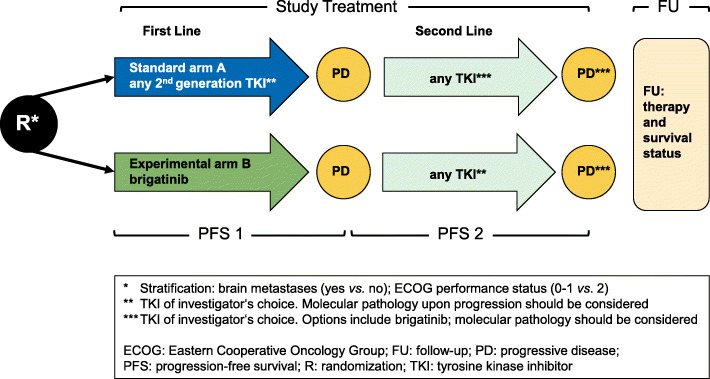
Study design. Eligible patients are randomized into the two treatment arms stratified by presence of brain metastases and ECOG performance status. First-line treatment in arm B is brigatinib, in arm A it is any other second-generation TKI of the investigator’s choice. All subsequent TKI will be chosen by the investigator and should take into account molecular tumor profiles upon progression. After the end of study treatment, patients will be followed-up for toxicity and survival. R: randomization, PD: progressive disease, TKI: tyrosine kinase inhibitor, FU: follow-up

Patients in standard arm A are treated in the first line with any approved second-generation TKI other than brigatinib (currently alectinib or ceritinib) according to the investigator’s choice, followed by second-line treatment with another approved ALK TKI of the investigator’s choice. Patients in the experimental arm B receive brigatinib in the first line, followed by any other approved ALK TKI in the second line according to the investigator’s choice (Fig. 1). It is recommended that the choice of second-line TKI takes into account the molecular tumor profile (e.g. presence of *ALK* resistance mutations) at the time of disease progression, which will be analyzed in tissue and liquid rebiopsies as part of the study and made available to the investigators using a “fast-track” procedure in order to support therapeutic decisions. Arm A patients failing standard second-generation TKIs will also have the option to receive brigatinib as second-line treatment, if so chosen by the treating physician, which will be provided by Takeda.

All TKI will be taken orally at the recommended daily doses, with predefined dose modifications in case of tolerability problems. Safety will be monitored by physical examinations, ECOG performance status, clinical laboratory profiles and continuous assessments of AE and QoL will further be evaluated using patient questionnaires (SF-12 and, in case of brain metastases, EORTC QLQ-BN20). Patients will be allowed to continue study treatment according to the decision of treating physicians for as long as they derive clinical benefit, i.e. radiologic progression per se does not necessitate a treatment switch.

#### Radiologic assessments

Baseline tumor evaluation will take place at screening by contrast-enhanced CT of the chest and abdomen. Tumor response will be evaluated according to RECIST 1.1 after two cycles (8 weeks) and every 12 weeks thereafter (Q12W ±7 days) in both the first and second line. Moreover, contrast-enhanced CT/MRI of the brain will be performed at baseline testing (MRI is strongly recommended by the study protocol). If intracranial lesions are detected, brain imaging is recommended with every scheduled tumor response assessment thereafter (intervals may be adapted by the investigators depending on the location and size of detected lesions), otherwise brain surveillance will take place with every second radiologic assessment, i.e. 20 weeks after beginning of first or second line, and every 24 weeks thereafter. Baseline disease assessment for the second therapy line must be performed within 30 days prior to treatment start. Assessments will continue until progression, death or initiation of another antineoplastic therapy after the two study treatment lines according to the standard of care (SOC).

#### Sampling for molecular profiling

Comprehensive clinical phenotyping and molecular tumor characterization are pivotal to this trial. Tumor biopsies are collected prior to the start of first-line treatment as formalin-fixed paraffin-embedded (FFPE) tissue. Moreover, blood samples will be taken at baseline and with every radiologic assessment during the first and second therapy line.

At the time of progression, “fast-track” NGS-based molecular profiling of liquid (ctDNA) and tissue rebiopsies (preferentially on new or growing lesions) will be offered to guide the choice of next-line therapy. In case of isolated intracranial progression, ctDNA-based NGS profiling using cerebrospinal fluid samples will be offered if the patient gives written consent.

An overview of all study procedures is presented in Table 2.

**Table 2:** Schedule of Study Assessments and Procedures

Assessment	Screening period			Study treatment period (1st -and 2nd-line treatment, respectively) *					Follow-Up
	Screening (up to 28 days prior to D1)			Cycle 1 ^**a**^		Cycle 2 ^**a**^	Cycle 3 to Cycle n ^**a**^	EOT ^**b**^	
Day / Frequency	D-28 to D0	D-14 to D0	D-7 to D0	D1	D8	D1	D1 or D15 alternating (Q6W ±3d)		Q12W ±21 days
Informed consent, eligibility criteria, demographics, medical / surgical history ^c^	X								
**Examinations**									
PE ^e^		X		X ^f,g^	X ^g,m^	X	X	X	
Height		X							
Vital signs ^o^, Weight			X	X ^f^	X	X	X	X	
ECG		X		Whenever clinically indicated				X	
ECOG Performance Status		X		X ^f^		X	X	X	
**Laboratory**									
Hematology ^p^			X	X ^f^		X	X	X	
Chemistry ^p^			X	X ^f^	X ^h^	X	X	X	
Coagulation ^p^			X						
Pregnancy test ^i^			X	X ^f^	Every 12 weeks (Q12W ±7 days)			X	
Cerebrospinal fluid (***local lab***) (optional)				Recommended for “brain-only” progression at time of PD					
**Study treatment**									
Arm A – 1st line: Any 2nd-generation TKI				Dose and frequency according to current SmPC					
Arm B – 1st line: Brigatinib				Once daily oral dose (QD p.o.) [90 mg D1 to D7, 180 mg from D8 ^g,m^]					
Arm A – 2nd line: Any TKI				Dose and frequency according to current SmPC					
Arm B – 2nd line: Any TKI				Dose and frequency according to current SmPC					
Diary review with patient ^k^				X ^k^		X	X	X	
**Continuous assessments**									
Disease assessment ^q^	X			Throughout study ^q^					X ^q^
QoL ^j^			X	Together with imaging of chest and abdomen				X	
AE / toxicities				Throughout study at every treatment day					
Concomitant medications				Throughout study					
**Biomarker sampling**									
Biopsy (FFPE tumor tissue) ^**d**^	X			After failure of first-line TKI ^d^ After failure of second-line TKI ^d^					
Blood sample collection ^n^			X	Throughout study ^n^				X	
Cerebrospinal fluid				Recommended for “brain-only” progression (at time of PD)					
**Follow-up assessments**									
Survival ^l^									X ^l^
Subsequent anticancer therapy									X

* Second-line treatment is started after a washout period of 3 days. Choice of next-line treatment should take into account molecular results of rebiopsies. Disease assessment, including baseline imaging for the second line, must be performed within 30 days before therapy start

^a^A cycle is defined as 28 days; acceptable window for regular visits: ±3 days, starting from cycle 2

^b^EOT visits 30 days (±7d) after the last dose of first- and second-line treatment (30 days safety follow-up)

^c^Medical history with details on any prior NSCLC therapy, including start and end date(s), best response(s). NOTE: Assay and sample type of prior *ALK* tests must be documented

^d^Exploratory biomarker studies: Central NGS-based multiplex analysis is mandatory for baseline FFPE-tissue biopsies and recommended for lesions growing under therapy

^e^Complete physical examination (PE) at screening and EOT; symptom-directed PE at any other time point (at investigator’s discretion)

^f^PE, ECOG performance status assessments, hematology, chemistry, and pregnancy tests are required at C1D1 (unless performed within 7 days before and no changes anticipated)

^g^Early pulmonary symptoms must be evaluated on C1D8 before increasing the brigatinib dose

^h^Aspartate aminotransferase / alanine aminotransaminase and total bilirubin only

^i^Pregnancy tests (urine or serum beta-human chorionic gonadotropin only, obligatory for women of childbearing potential) must be confirmed negative within 7 days before TKI start (i.e., 7 days prior to C1D1). To be repeated once every 12 weeks thereafter (every 3 cycles; Q12W ±7 days) and at EOT visit (additional testing if indicated)

^j^QoL assessments at baseline, all tumor assessment visits (refer to footnote q), and at EOT visit (SF-12, and, in case of brain involvement, also EORTC QLQ-BN20)

^k^Patients must document TKI intake. On C1D8, on D1 of each cycle and at the EOT visit, site staff reviews diary and crosschecks tablets or tablet bottles in the patient’s possession

^l^Survival follow-up shall be scheduled Q12W ± 21 days until the end of the study

^m^Patients tolerating the brigatinib starting dose (90 mg, C1D1-D7) should increase the dose to 180 mg QD from C1D8

^n^Blood samples at baseline (i.e., up to 7 days before TKI start) and with every CT/MRI assessment during active first- and second-line therapy (i.e. two cycles [8 weeks] after TKI start and every 12 weeks thereafter [Q12W ±7 days] (at every CT/MRI assessment visit). Samples: 2 x 7.5 ml EDTA blood and 1x 7.5 ml serum. In addition, 2 × 2.5 ml PAXgene blood for RNA isolation at four time points: i.e., (1) up to 7 days before start of first-line TKI, (2) following five cycles of first-line TKI [at the second CT/MRI assessment], (3) up to 7 days before start of second-line TKI, (4) after five cycles of second-line TKI [with CT/MRI assessment]

^o^Vital signs: body temperature, pulse, blood pressure, oxygen saturation. Special attention must be paid to blood pressure monitoring at screening and during treatment. Patients with controlled hypertension at inclusion must receive appropriate antihypertensive treatment during study participation (antihypertensive medication at the investigator's discretion, to be documented in the subject’s Concomitant Medication eCRF)

^p^Laboratory assessments may be performed up to one day before the visit (entire study) in order to have results available on the visit day. Hematology panel: Complete Blood Cell (CBC) count with differential. Chemistry panel: albumin; alkaline phosphatase; alanine aminotransferase; aspartate aminotransferase; serum lipase, serum amylase; lactate dehydrogenase; creatine kinase; creatinine; calcium; glucose; phosphate; potassium; sodium; magnesium; total bilirubin; blood urea nitrogen or blood urea; C-reactive protein; gamma-glutamyltransferase. Coagulation: INR or PTT only at baseline

^q^Contrast-enhanced CT/MRI (chest/abdomen): all patients (unless contraindicated). Tumor response is evaluated according to RECIST 1.1 (investigator assessment). Baseline tumor evaluation at screening; response assessment according to SOC, i.e. after two cycles (8 weeks) and every 12 weeks thereafter (Q12W ±7 days) during active treatment in the first and second line. Second-line: baseline disease assessment should be performed within 30 days before TKI start. Intracranial response evaluation according to RECIST 1.1 (all patients): contrast-enhanced brain MRI/CT at screening (MRI strongly recommended). In case of brain metastasis, imaging is recommended with every scheduled assessment (intervals may be adapted depending on location and size of lesions), otherwise brain surveillance should take place according to SOC (with every second radiologic assessment). Assessments will continue until progression, death or initiation of another antineoplastic therapy after the two study treatment lines according to SOC

Study management and data quality assurance will be conducted following the standard operational procedures of the *Institut für Klinische Krebsforschung IKF GmbH am Krankenhaus Nordwest* (Frankfurt, Germany). An eCRF for data collection will be carefully maintained for each participant, also reporting serious and non-serious AE according to the common criteria for adverse events (CTCAE, version 5.0), and changes of study treatment throughout the trial. Patients will be provided a diary where the study drug intake will be recorded. Missed doses will be captured by the patient diaries and documented in the eCRF.

After the end of the study period, participants will be proactively followed up for survival and/or treatment-related AE until loss-to-follow-up or withdrawal of study consent. Patients unwilling to return to the study site will alternatively be offered a follow-up by telephone every three months. The treating physicians will ensure that patients receive appropriate further therapies according to SOC.

### Statistical analysis

Statistical analysis will follow the International Conference on Harmonization (ICH) Guidelines “Structure and Content of Clinical Study Reports” and “Statistical Principles for Clinical Trials”.

#### Sample size and study duration

The sample size of *n* = 116 is primarily determined by considerations of enrollment feasibility. The planned study duration was based on published data from the ALTA-1 L [12] and ALEX [32, 33] phase III trials. In these calculations, the HR for PFS of first-line alectinib vs. crizotinib was set to 0.45 (i.e. the average of 0.47 [32] and 0.43 [33], the HR for PFS of first-line brigatinib vs. crizotinib was assumed identical to that of alectinib vs. crizotinib (based on data of the ALTA-1 L trial with HR = 0.49, 95% confidence intervall (CI) 0.33–0.74 [12]), and the median PFS under first-line crizotinib was considered to be 11 months (i.e. the average of 11.1 [32] and 10.9 months [33]). Consequently, assuming an exponential distribution of PFS, the expected median PFS under first-line alectinib or brigatinib was estimated as 24.4 months (11/0.45), and the proposed duration of the ABP trial was based on an expected total follow-up time of 32 months for the last patient, i.e. an expected first-line PFS of 24.4 months plus an expected second-line PFS of approximately 7 months (based on the median PFS of 5.5–6.9 months [95% CI 2.9–9.5] under lorlatinib after failure of second-generation ALK inhibitors in the EXP3B/4/5 cohorts of a phase II trial [34]). The recruitment period of 36 months was proposed based on the expected number of newly diagnosed ALK^+^ patients in the centers expected to participate.

To quantify the potential degree of evidence regarding PFS1 that can be gained with a total of 116 patients, we calculated the number of expected events *d*, the expected 95% CI for the median PFS of alectinib and brigatinib in the first line (assumed to be equal, as explained above), and the expected 95% CI for the HR of PFS under first-line brigatinib vs. alectinib in the ABP trial, given a constant accrual over a time of 36 months, a follow-up time of 32 months for the last patient, and exponentially distributed PFS times. Under these assumptions, the expected number of PFS events is *d* = 87, the expected 95% CI of the median PFS in the first line is [16.6–34.2 months] (both arms), and the expected 95% CI of the HR for PFS in the first line [0.66–1.52]. The number of events *d* was calculated using Schoenfeld’s formula [35] and ADDPLAN v6.1 software. The CI for the median PFS was calculated via bootstrapping using 1,000,000 datasets simulated in R v3.3.3 (http://r-project.org) and a fixed random number seed to yield stable and reproducible results, and the CI for the HR was calculated using the (approximate) formula exp. (±1.96√4/d) [36].

#### Methods of statistical analysis

A Cox proportional hazards model will be used to assess the primary endpoint PFS1. As covariates, the model includes the factor “treatment group” and is adjusted for the presence of brain metastases (yes vs. no) and ECOG (0–1 vs. 2) at baseline. The treatment groups will be compared at a two-sided α of 0.05, and a 95% CI for the HR determined. Furthermore, Kaplan-Meier curves will be provided. Primary analysis will be based on all randomized patients (intention-to-treat population), while sensitivity analyses will be conducted for the per-protocol set (patients without major protocol violations) and for predefined subgroups of secondary and exploratory endpoints.

Analyses of secondary endpoints will be descriptive and will include the calculation of appropriate summary measures of the empirical distributions. AE and SAE will be summarized by relative and absolute frequency and severity grade based on CTCAE V5.0. Summary tables will provide the number and percentage of patients with AE and the 95% CI for the event rates.

A solely descriptive interim evaluation to assess the primary (PFS of first-line treatment) and secondary (iORR, iDOR, iTTP, TNT of first line treatment, PFS of second-line treatment, TNT of second line treatment, OS, QoL, frequency of SAE) endpoints will be performed when 25 patients in each arm have a follow-up of at least 12 months.

All analyses will be carried out using SAS (SAS Institute, Cary, NC) version 9.4 or higher.

### Trial status

As of June 21, 2021, 25 study centers have been initiated. The first patient was enrolled on June 18, 2020.

## Discussion

The ABP trial has been designed to provide insights and generate hypotheses that will facilitate optimization of management for patients with ALK^+^ NSCLC. Besides supporting therapeutic decisions for enrolled patients by providing “fast-track” molecular results to the treating physicians, the ABP trial also aims to deepen understanding of the underlying biology and facilitate development of a framework for individualized management of ALK^+^ NSCLC according to molecular properties. One special focus is the relationship of resistance mechanisms acquired under treatment with the therapeutic compound used and baseline tumor characteristics. Patients with low molecular risk and the perspective of a “chronic disease” will be distinguished from “high-risk” cases, and molecular features of the latter will be utilized to elaborate improved non-invasive methods of disease monitoring and preclinical modeling in order to advance therapeutic strategies.

## Data Availability

Data generated by this study will be made available by the corresponding author on reasonable request.

## Abbreviations

AE: Adverse event
ALK: Anaplastic lymphoma kinase
CI: Confidence interval
CNS: Central nervous system
CT: Computed tomography
CTCAE: Common Terminology Criteria for Adverse Event
ctDNA: Circulating tumor DNA
DOR: Duration of response
ECOG: Eastern Cooperative Oncology Group
eCRF: Electronic case report form
EGFR: Epidermal growth factor receptor
EML4: Echinoderm microtubule-associated protein-like 4
EOT: End of treatment
FFPE: Formalin-fixed, paraffin embedded
HR: Hazard ratio
ICH: International Council for Harmonisation
iDoR: Intracranial duration of response
iORR: Intracranial objective response rate
KRAS: Kirsten rat sarcoma viral oncogene homolog
MRI: Magnetic resonance imaging
NGS: Next-generation sequencing
NSCLC: Non-small cell lung cancer
OS: Overall survival
PFS: Progression-free survival
PE: Physical examination
QoL: Quality of life
RECIST 1.1: Response Evaluation Criteria in Solid Tumors, version 1.1
SAE: Serious adverse event
SmPC: Summary of Product Characteristics
SOC: Standard of care
TKI: Tyrosine kinase inhibitor
TNT: Time to next treatment
TP53: Tumor Protein 53
iTTP: Time to intracranial progression
